# General Practitioner Sex and Documented Cancer Prevalence in German Primary Care: A Matched Cross-Sectional Study

**DOI:** 10.3390/epidemiologia7050146

**Published:** 2026-10-19

**Authors:** Karel Kostev, Isabel Klein, Svetlana Alymova, Marcel Konrad

**Affiliations:** 1 Marburg University, University Hospital, 35043 Marburg, Germany; 2 Epidemiology, IQVIA, 60549 Frankfurt am Main, Germany; 3 Real-World Data Analytics, IQVIA, Real-World Data Analytics, 60549 Frankfurt am Main, Germanysvetlana.alymova@iqvia.com (S.A.); 4 Health & Social, FOM University of Applied Sciences for Economics and Management, 60486 Frankfurt am Main, Germany

**Keywords:** general practice, physician sex, cancer, primary care, skin cancer, Germany

## Abstract

Background/Objectives: General practitioner’s (GP) sex has been associated with differences in consultation behaviour, preventive care, and physical examination practices. It remains unclear whether these differences translate into variation in documented cancer diagnoses in primary care. This study examined associations between GP sex and documented cancer prevalence across a broad range of malignancies in Germany. Methods: This retrospective cross-sectional study used data from the IQVIA™ Disease Analyzer database (IQVIA, Frankfurt am Main, Germany), a representative German outpatient database. Individuals aged 18 years and older who attended one of 1182 general practices in 2025 were assigned to the practice they consulted and matched 1:1 by propensity score on patient sex, age, insurance type, and practice size. Thirteen ICD-10 cancer categories were analysed, with GP sex as the independent variable (male GP sex as the reference category), stratified by patient sex, using generalized estimating equations (GEEs) with the practice as the clustering unit and cluster-robust standard errors as the primary analysis; generalized linear mixed models with a practice-level random intercept were fitted as a sensitivity analysis. Absolute prevalence differences per 1000 patients were estimated with cluster-robust linear models. Two-sided *p*-values < 0.05 were considered statistically significant, and Bonferroni and Benjamini–Hochberg false discovery rate (FDR) corrections were applied to a pre-specified family of 23 models. Results: A total of 1,204,254 individuals seen by female GPs (mean age 53.3 [standard deviation, SD, 19.8] years; 54.8% female) and 1,204,254 individuals seen by male GPs (mean age 53.7 [SD 19.8] years; 55.2% female) were included. In the cluster-adjusted (GEE) models, female GP sex was associated with higher odds of documented skin cancer in female patients (odds ratio [OR] 1.23, 95% confidence interval [CI] 1.11–1.35; absolute difference 1.15 per 1000 patients, 95% CI 0.58–1.73), which remained significant after both FDR and Bonferroni correction; in male patients the association was weaker and did not remain significant after correction (OR 1.14, 95% CI 1.03–1.26). In exploratory analyses, the association was confined to non-melanoma skin cancer (female patients OR 1.27, 95% CI 1.14–1.42) and to patients aged 35 years and older. The inverse association for mesothelial and soft tissue cancer seen in unadjusted models was no longer statistically significant after accounting for practice-level clustering (female patients OR 0.75, 95% CI 0.43–1.31; male patients OR 0.75, 95% CI 0.45–1.25). No statistically significant associations were observed for breast, digestive, genital organ, haematological, or other cancer types. Conclusions: After accounting for clustering at the practice level, female GP sex was associated with a higher prevalence of documented skin cancer—most robustly among female patients—whereas associations for other malignancies, including mesothelial and soft tissue cancer, were not statistically significant. The design cannot distinguish differences in examination from differences in documentation. These findings have implications for the interpretation of routine cancer data and indicate that practice-level factors, and the clustering of patients within practices, should be considered when documented cancer prevalence is analysed in primary care data.

## 1. Introduction

In primary care, the pathway to a cancer diagnosis is a multi-step process that typically extends beyond a single clinical encounter. It can be separated into four stages: entry into the diagnostic cascade, referral to a specialist, diagnostic confirmation, and documentation of the confirmed diagnosis in the primary care record. General practitioners (GPs) often play a coordinating role in oncological care pathways: they are commonly the first point of contact for patients presenting with symptoms, they initiate preliminary diagnostic work-up, including laboratory investigations, and they refer patients to specialists or inpatient facilities when malignancy is suspected [1,2]. In many cancer cases, however, the definitive diagnosis is established not in primary care but by specialists or in hospital settings. The confirmed diagnosis is then communicated back to the GP through discharge summaries or specialist correspondence and documented in the primary care record [3,4]. Accordingly, for most cancer entities, a GP-recorded diagnosis may reflect an externally confirmed finding rather than an independent diagnostic act.

Given this largely coordinative and documentary role, one might expect differences in GP-level documented cancer prevalence to be smaller than for conditions primarily diagnosed and managed within primary care. Physician sex has nevertheless been linked to consultation length, breadth of physical examination, communication about screening and prevention, and the delivery of preventive care across a range of clinical contexts and health-care systems [5,6,7,8,9,10]. Moreover, GPs do not merely record externally established diagnoses; they also shape entry into the diagnostic cascade through decisions to examine beyond the presenting complaint, to order tests, and to refer [11]. Whether such differences translate into measurable variation in documented cancer prevalence remains unclear, and any observed difference may equally reflect patient mix, care pathways, or recording practices.

The German primary care setting provides a particularly relevant context for examining this question. In Germany, GPs act as generalists, manage a broad spectrum of conditions, and maintain longitudinal patient records. Skin cancer is of particular relevance in this context: non-melanoma skin cancer is the most frequently diagnosed malignancy in Germany, and malignant melanoma is among the most common cancers in both sexes [12]. For certain cancer entities—most notably skin cancer—GPs may themselves identify suspicious findings during routine consultations, in addition to documenting diagnoses communicated by dermatologists or other specialists [13,14]. In the German system, patients may also consult dermatologists directly for skin cancer screening without GP referral [15,16]. As a result, GP-documented skin cancer diagnoses may enter the primary care record through multiple pathways, including independent clinical observation, active referral, and subsequent documentation of specialist findings [17].

Across the broader landscape of cancer diagnoses, it remains unclear whether male and female GPs differ in the prevalence of documented malignancies in their patient panels, and whether any such differences are consistent across cancer types or confined to specific entities [18]. When routinely collected cancer data are interpreted, several sources of variation must be considered, including patient case-mix, completeness and accuracy of documentation, coding practices, access pathways, and the clustering of patients within practices [19].

The aim of this study is to compare the prevalence of documented cancer diagnoses across a broad range of malignancies between male and female GPs in Germany using routinely collected primary care data. The research gap addressed here is that no large-scale study has examined documented cancer prevalence by GP sex across the full spectrum of malignancies while accounting for the clustering of patients within practices. We hypothesise that female GP sex is associated with a higher documented prevalence of those malignancies that GPs can identify themselves, most notably skin cancer, and with no difference for malignancies whose diagnosis is established upstream of the GP encounter.

## 2. Materials and Methods

### 2.1. Data Source

This study was based on data from the IQVIA™ Disease Analyzer database. This database contains anonymized longitudinal data on diagnoses, prescriptions, and patient demographics from general and specialist practices in Germany and has been shown to be broadly representative of major chronic diseases and patient characteristics in the German primary care setting [20]. Data are transmitted monthly from practice management systems via encrypted, standardized interfaces, thereby reflecting routine clinical records. The database includes approximately ten million patients from around 2500 practices across eleven medical specialties. Sampling is based on annual statistics from the German Medical Association and stratified by specialist group, federal state, community size, and physician age. The database has been widely used in epidemiological research, including studies on cancer in Germany [21].

### 2.2. Study Design and Population

**Figure 1:**
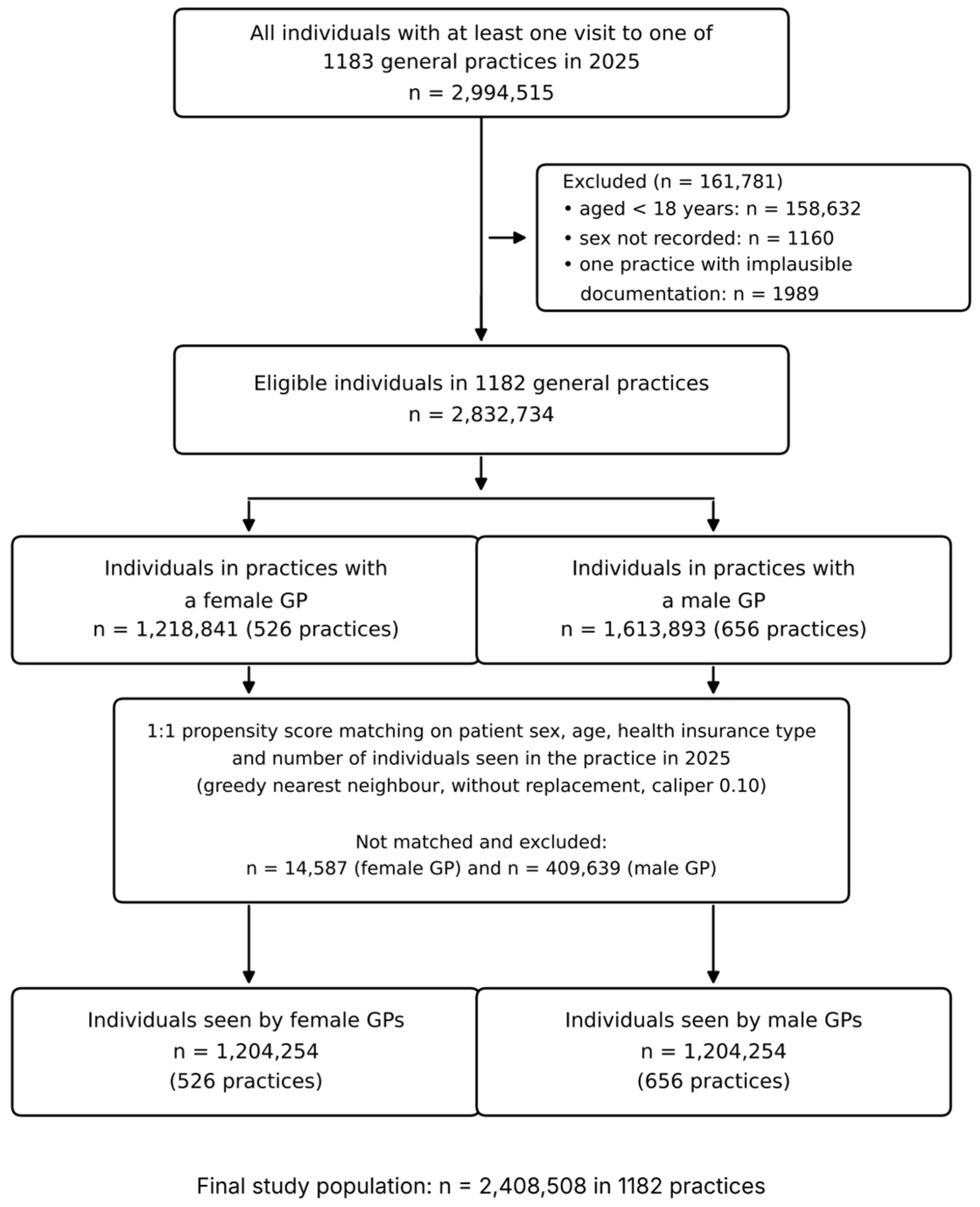
Selection of study population. Propensity score matching was based on patient sex, age, health insurance type, and the number of individuals seen in the practice in 2025.

This retrospective cross-sectional study included all individuals aged 18 years and older with at least one visit to one of 1182 general practices in 2025; individuals with unknown sex were excluded, as was one practice with implausible documentation. The unit of observation in the database is the practice rather than the individual physician, and the sex recorded for a practice is that of the practice owner. Practices were therefore classified by the sex of the practice owner, and patients were assigned to the practice they consulted. In single-physician practices the practice owner is necessarily the physician who documents the diagnosis. In group practices, which may comprise physicians of both sexes, the documenting physician may be another member of the practice, so that exposure can be misclassified; this misclassification is non-differential with respect to the outcome and biases estimates towards the null. A sensitivity analysis was therefore restricted to single-physician practices, in which the assignment of GP sex is unambiguous. Patients are recorded longitudinally within one practice and cannot be linked across practices, so each individual contributes exactly one record per calendar year to exactly one exposure group, irrespective of the number of consultations during the year. No minimum panel size was applied; practice size ranged from 60 to 18,860 individuals in 2025. To ensure comparability between patient groups, individuals seen in 2025 by female and male GPs were matched 1:1 using nearest-neighbour propensity score matching based on patient sex, age (in years), health insurance type (statutory vs. private), and the number of individuals seen in the practice in 2025. Matching was performed without replacement using a greedy algorithm within deciles of the propensity score, with a caliper of 0.10 standard deviations of the logit of the propensity score; unmatched individuals were excluded. The propensity score was intentionally defined at the patient level to balance the demographic case-mix of the two patient groups; the dependence between patients of the same practice was instead addressed in the analysis by accounting for the clustering of patients within practices (Section 2.3). Practice characteristics beyond practice size were deliberately not included in the propensity score, because balancing them at the patient level would not remove the dependence between patients of the same practice. Covariate balance after matching was assessed using standardized mean differences, with values below 0.1 considered indicative of adequate balance [22]. The patient selection process is illustrated in Figure 1.

### 2.3. Study Outcomes and Statistical Analysis

The primary outcome was the association between GP sex and the prevalence of documented cancer diagnoses. For each cancer type, the prevalence was calculated as the number of individuals with a confirmed or status-post cancer diagnosis in 2025 divided by the total number of individuals seen by the respective GPs in that year, and expressed per 1000 patients. German practice management systems record a diagnosis-certainty qualifier that distinguishes confirmed diagnoses (gesicherte Diagnose) from status-post entries (Zustand nach). Both were included in the primary outcome definition, and a sensitivity analysis was restricted to confirmed diagnoses. Throughout, a documented diagnosis denotes a diagnosis recorded in the primary care record, irrespective of where it was established.

Because patients were nested within 1182 practices, the association between GP sex (female vs. male, male GP sex as the reference category) and the presence of each documented cancer diagnosis was estimated using generalized estimating equations (GEEs) with a binomial distribution, a logit link and the practice as the clustering unit, an independent working correlation structure, yielding empirical (cluster-robust) standard errors [19]; this was the primary analysis, and generalized linear mixed models with a practice-level random intercept estimated by Laplace approximation were fitted as a sensitivity analysis. An odds ratio above 1 indicates higher odds of a documented diagnosis among patients of female GPs. Absolute prevalence differences per 1000 patients with cluster-robust 95% confidence intervals were estimated from linear models with an identity link on the same matched data. Analyses were conducted separately for female and male patients to account for sex-specific cancer patterns. Cancer types were classified according to ICD-10 codes and included: lip, oral cavity and pharynx (C00–C14); digestive organs (C15–C26); respiratory and intrathoracic organs (C30–C39); bone and articular cartilage (C40–C41); skin (C43–C44); mesothelial and soft tissue (C45–C49); female breast (C50); female genital organs (C51–C58); male genital organs (C60–C63); urinary tract (C64–C68); central nervous system (C69–C72); thyroid and other endocrine glands (C73–C75); and lymphoid, haematopoietic and related tissue (C81–C96). Four sensitivity analyses were specified: restriction to confirmed diagnoses; restriction to single-physician practices, in which the assignment of GP sex is unambiguous; restriction to practices with at least 500 individuals in 2025; and additional adjustment for healthcare utilisation, operationalised as the number of documented consultation days per patient in 2025. In addition, two exploratory analyses were performed for skin cancer: separate estimation for melanoma (C43) and non-melanoma skin cancer (C44), and stratification by the age threshold of 35 years, at which entitlement to statutory skin cancer screening begins.

Results are reported as odds ratios (ORs) with 95% confidence intervals (CIs), absolute prevalence differences per 1000 patients, and corresponding *p*-values. The pre-specified testing family comprised 23 models: ten cancer categories analysed in both female and male patients, female breast cancer and female genital organ cancers in women only, and male genital organ cancers in men only. A Bonferroni correction, multiplying each raw *p*-value by 23, and a Benjamini–Hochberg false discovery rate (FDR) correction applied to the same family of 23 ranked *p*-values were used, and raw, FDR-adjusted, and Bonferroni-adjusted *p*-values are reported for every model. The sensitivity and exploratory analyses lie outside this family, are reported without further correction, and are interpreted descriptively. All analyses were performed using SAS version 9.4 (SAS Institute, Cary, NC, USA).

## 3. Results

### 3.1. Baseline Characteristics

A total of 1,204,254 individuals seen by female GPs and 1,204,254 individuals seen by male GPs were included after 1:1 propensity score matching, resulting in a final study population of 2,408,508 patients. Before matching, 1,218,841 individuals attended practices with female GPs and 1,613,893 attended practices with male GPs, meaning that 14,587 and 409,639 individuals, respectively, remained unmatched; all 1182 practices remained represented after matching. Baseline characteristics after matching are presented in Table 1. After matching, covariate balance between groups was achieved, with all standardized mean differences below 0.1 for the matching variables. Mean age was 53.3 (SD 19.8) years in the female GP group and 53.7 (SD 19.8) years in the male GP group. The proportion of female patients was 54.8% and 55.2%, respectively. The majority of patients in both groups held statutory health insurance (92.0% vs. 91.5%). The median number of individuals seen in the practice in 2025 was 2564 (IQR 1688) for female GPs and 2526 (IQR 1648) for male GPs. At the practice level, the median number of individuals per practice was 2096 (IQR 1532-2859), and the number of physicians working in a practice ranged from one to seven. Of the 1182 practices, 944 (79.9%) were single-physician practices; this was more frequent among practices with male GPs (585 of 656, 89.2%) than among practices with female GPs (359 of 526, 68.3%).

### 3.2. Crude Cancer Prevalence by GP Sex

**Figure 2:**
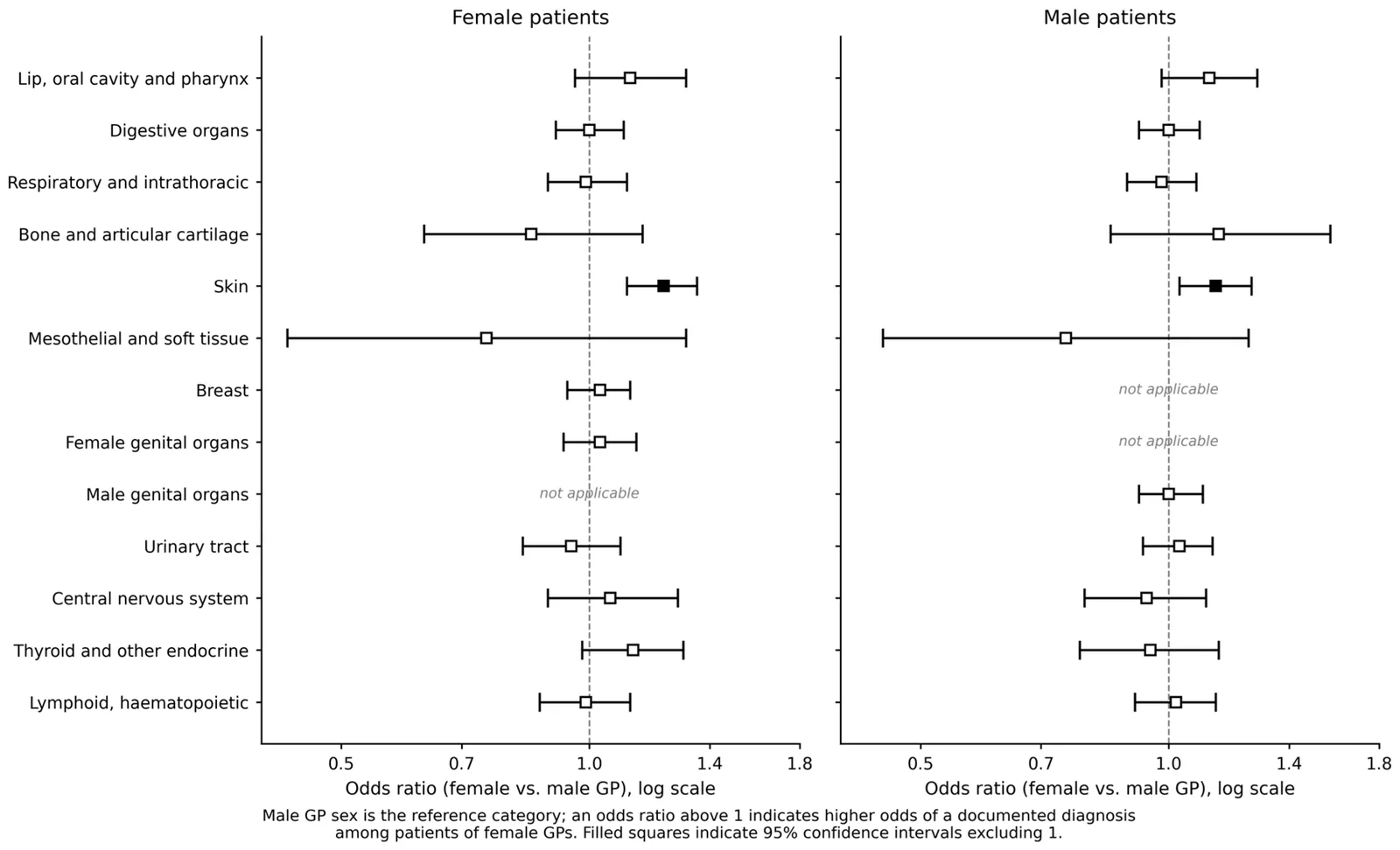
Cluster-adjusted odds ratios (female vs. male GP) with 95% confidence intervals for documented cancer diagnoses, by patient sex.

Crude (unadjusted) documented cancer prevalence per 1000 patients, stratified by GP sex and patient sex, is summarised below; adjusted associations are shown in Figure 2 and Table 2. Among female patients, the most prevalent cancer entities were breast cancer (11.4 per 1000 for female GPs vs. 11.1 per 1000 for male GPs), followed by skin cancer (6.3 vs. 5.1), and cancers of digestive organs (4.8 vs. 4.8). Among male patients, cancers of the male genital organs were most prevalent (10.2 per 1000 for both GP sexes), followed by cancers of digestive organs (6.8 vs. 6.8) and skin cancers (6.6 vs. 5.8). Across both patient sexes, descriptively higher skin cancer prevalence was observed among patients seen by female GPs compared with those seen by male GPs. The corresponding absolute differences were 1.15 per 1000 patients (95% CI 0.58–1.73) in female patients and 0.79 per 1000 patients (95% CI 0.18–1.40) in male patients; absolute differences for all cancer categories are reported in Table 2. Differences for other entities, including mesothelial and soft tissue cancer, were small in absolute terms; as shown below, several of these crude differences did not persist once practice-level clustering was taken into account.

### 3.3. Association Between GP Sex and Documented Cancer Diagnoses

Results of the cluster-adjusted (GEE) analyses are presented in Table 2 and Figure 2. After accounting for clustering at the practice level, one association remained statistically significant after both FDR and Bonferroni correction: skin cancer in female patients, for which female GP sex was associated with higher odds of a documented diagnosis (OR 1.23, 95% CI 1.11–1.35; raw *p* < 0.001; FDR *p* = 0.002; Bonferroni *p* = 0.002; absolute difference 1.15 per 1000 patients, 95% CI 0.58–1.73).

In male patients, skin cancer showed a directionally consistent but weaker association that did not remain significant after multiple-testing correction (OR 1.14, 95% CI 1.03–1.26; raw *p* = 0.011; FDR *p* = 0.130; absolute difference 0.79 per 1000 patients, 95% CI 0.18–1.40).

The inverse association for mesothelial and soft tissue cancer that had been observed in unadjusted models was no longer statistically significant once clustering was accounted for, with markedly wider confidence intervals reflecting the rarity of these tumours and their concentration within relatively few practices (female patients OR 0.75, 95% CI 0.43–1.31, *p* = 0.31; male patients OR 0.75, 95% CI 0.45–1.25, *p* = 0.27). The 1788 documented cases of this category occurred in 713 of the 1182 practices, and a single practice accounted for 272 of them.

No other cancer type showed a statistically significant association with GP sex (Table 2). The mixed-model sensitivity analysis supported these conclusions: skin cancer in female patients remained significant (OR 1.23, 95% CI 1.12–1.35), skin cancer in male patients was significant in the uncorrected model (OR 1.15, 95% CI 1.05–1.27), and mesothelial/soft tissue cancer was null (OR ≈ 1.0). The practice-level random-intercept variance was clearly non-zero across models (0.44 for skin cancer and 0.75 for mesothelial and soft tissue cancer, corresponding to intraclass correlation coefficients (ICCs) of 0.12 and 0.18 and to median odds ratios (MORs) of 1.88 and 2.28), confirming meaningful between-practice heterogeneity.

Three of the four sensitivity analyses did not change these conclusions. When the analysis was restricted to confirmed diagnoses, the skin cancer association was OR 1.21 (95% CI 1.08–1.35) in female and OR 1.13 (95% CI 1.01–1.25) in male patients; status-post entries accounted for 18.2% and 19.9% of all C44 entries in the male and female GP groups, respectively. When the analysis was restricted to practices with at least 500 individuals in 2025, which excluded 14 practices and 2803 individuals, the estimates were OR 1.22 (95% CI 1.11–1.35) and OR 1.14 (95% CI 1.03–1.26). With additional adjustment for the number of consultation days per patient, they were OR 1.20 (95% CI 1.08–1.33) and OR 1.11 (95% CI 1.01–1.23).

The fourth sensitivity analysis, restricted to the 944 single-physician practices in which the sex of the documenting physician is unambiguous, comprised 932,637 female and 770,981 male patients, that is, 71% of the matched population. In this subset, the association was stronger than in the main analysis: OR 1.27 (95% CI 1.12–1.44) in female and OR 1.16 (95% CI 1.03–1.30) in male patients. The separation of the two skin cancer entities was also more pronounced, with OR 1.36 (95% CI 1.18–1.56) for non-melanoma skin cancer and OR 1.04 (95% CI 0.90–1.21) for melanoma in female patients, and OR 1.18 (95% CI 1.04–1.35) and OR 1.09 (95% CI 0.93–1.28), respectively, in male patients. For mesothelial and soft tissue cancer the estimates were OR 0.87 (95% CI 0.71–1.06) in female and OR 1.01 (95% CI 0.82–1.24) in male patients, with markedly narrower confidence intervals than in the main analysis.

In the exploratory analyses, the skin cancer association was carried by non-melanoma skin cancer. In female patients the odds ratio was 1.27 (95% CI 1.14–1.42) for C44 and 1.12 (95% CI 0.99–1.27) for C43; in male patients it was 1.15 (95% CI 1.03–1.28) and 1.12 (95% CI 0.98–1.28), respectively. In single-physician practices the contrast was more pronounced, with 1.36 (95% CI 1.18–1.56) for C44 and 1.04 (95% CI 0.90–1.21) for C43 in female patients. Stratification by the 35-year age threshold of the statutory skin cancer screening programme showed the association only among individuals entitled to screening: OR 1.23 (95% CI 1.12–1.36) in women and OR 1.15 (95% CI 1.05–1.27) in men aged 35 years and older, compared with OR 1.03 (95% CI 0.70–1.52) and OR 0.96 (95% CI 0.57–1.62) below 35 years.

## 4. Discussion

This large cross-sectional study, drawing on routinely collected primary care data from over 2.4 million matched patients in Germany, examined associations between GP sex and the prevalence of documented cancer diagnoses across a broad range of malignancies. After accounting for the clustering of patients within 1182 practices, one association was statistically significant after both FDR and Bonferroni correction: patients registered with female GPs had a higher prevalence of documented skin cancer, an effect that was robust among female patients and directionally consistent, though weaker and not correction-significant, among male patients. An inverse association for mesothelial and soft tissue cancer that was apparent in unadjusted models did not persist once practice-level clustering was taken into account. For all other cancer entities, including breast cancer, male genital organ cancers, and digestive organ cancers, no significant associations with GP sex were observed.

The importance of accounting for practice-level clustering is itself a key methodological finding of this study. Patients seen by the same GP share exposure to that physician’s examination, referral, and documentation practices, so their outcomes are correlated; ignoring this dependence underestimates standard errors. In our data the practice-level random-intercept variance was clearly non-zero (intraclass correlation coefficient 0.12 for skin cancer and 0.18 for mesothelial and soft tissue cancer, corresponding to median odds ratios of 1.88 and 2.28), and moving to cluster-robust models widened the confidence intervals appreciably. This was inconsequential for the skin cancer association in female patients, which is based on a comparatively common outcome and remained robust, but it was decisive for the mesothelial and soft tissue category—a rare outcome concentrated within relatively few practices, with a single practice accounting for 272 of 1788 documented cases—where the apparent association disappeared. This illustrates that failure to account for clustering produces overly narrow confidence intervals and misleading statistical inference for rare, practice-concentrated diagnoses in real-world cancer data, and argues for routinely accounting for such clustering in analyses of documented cancer prevalence [19].

The most robust finding of this study is a positive association between female GP sex and higher documented skin cancer prevalence, most clearly observed in female patients. To our knowledge, this is the first study to report such an association in a large-scale primary care dataset. Because the sex recorded for a practice is that of the practice owner, exposure is unambiguous only in single-physician practices. The association was stronger there than in the whole population, which suggests that the main analysis underestimates the difference as a consequence of non-differential exposure misclassification in group practices. The association does not establish that female GPs diagnose more skin cancers, screen more frequently, or detect cancer more reliably; differences in recording practices, referral patterns, patient characteristics not captured by matching, and other practice-level factors are equally compatible with the observation. Several mechanisms may contribute to this observation, none of which were measured directly in this study.

First, skin cancer is one of the few malignancies for which GPs in Germany are not solely dependent on specialist referrals for diagnosis. Under the statutory skin cancer screening programme, GPs are qualified to perform visual skin examinations and may themselves identify suspicious lesions, in addition to documenting diagnoses received from dermatologists [15,16,17]. Prior research from France has shown that female GPs deliver preventive care, including physical examinations, more consistently than their male colleagues [8]. Two of our exploratory findings are compatible with this interpretation without confirming it: the association was confined to non-melanoma skin cancer, the entity most likely to be identified during a routine visual examination, and it was present only among individuals aged 35 years and older, that is, among those entitled to statutory skin cancer screening.

However, an alternative and equally plausible explanation concerns documentation rather than detection. Because patients in Germany can consult dermatologists directly for skin cancer screening without a GP referral, a substantial proportion of skin cancer diagnoses in the GP record may originate from specialists. Higher documented skin cancer prevalence among patients of female GPs may therefore reflect more complete and diligent recording of externally established diagnoses rather than superior detection by the GPs themselves. Our data cannot distinguish these mechanisms, and we present detection and documentation as two non-exclusive explanations.

A further, specifically German mechanism may contribute to this pattern. The statutory skin cancer screening is reimbursed extra-budgetarily through a dedicated billing code for patients aged 35 years and older every two years and can be billed together with the general health check-up within the same consultation; a greater propensity of female GPs to perform and bill these preventive services could therefore contribute to the higher documented skin cancer prevalence in their panels. The age pattern reported above is consistent with this mechanism, but the billing data themselves could not be examined here, because the cohort is defined at the practice level, the corresponding screening codes in the database are recorded on the dermatology side, and the data do not permit linkage of individual patients across practices or specialties. Quantifying the joint billing of these preventive services by GP sex is an important direction for future research using linked reimbursement data.

Second, patient behaviour may contribute to this pattern. Evidence suggests that patients—particularly women—prefer to consult a physician of the same sex for examinations involving undressing or inspection of skin areas that may be perceived as intimate [23,24], and such a preference would not be captured by propensity score matching on demographic variables alone.

Third, the association was observed in both female and male patients, which argues against an explanation based solely on sex concordance between patient and physician. The fact that male patients of female GPs also showed higher skin cancer prevalence may suggest that the association reflects a broader characteristic of how female GPs approach skin examination or referral behaviour, rather than a preference-driven effect limited to female patients. This is consistent with findings from Delpech et al. [8], who reported that female GPs perform preventive care more frequently regardless of patient sex, and with the broader literature documenting longer consultation times and more thorough clinical assessments among female physicians [5,6].

In our original, unclustered analysis we had observed an inverse association between female GP sex and documented mesothelial and soft tissue cancer, and had considered whether differences in patients’ occupational histories might explain it. After appropriate adjustment for practice-level clustering, this association was no longer statistically significant, with wide confidence intervals consistent with the rarity of these tumours (ICD-10 C45–C49) and with their concentration in few practices. We therefore no longer interpret this category as showing a difference by GP sex, and we do not attribute it to occupational or other exposures. This entity illustrates the sensitivity of rare-outcome estimates to the modelling of clustering.

The absence of significant associations for the majority of cancer types is itself an informative finding. For cancer entities whose diagnoses are typically established through specialist or hospital workup—including digestive organ cancers, respiratory cancers, haematological malignancies, and male or female genital organ cancers—GP sex was not associated with differences in documented prevalence. This is consistent with the hypothesis that, for these cancer types, the GP’s role is primarily one of documentation and coordination rather than independent detection. When the diagnostic act lies predominantly upstream of the GP encounter, physician-level variation in examination behaviour or referral patterns may have limited influence on what ultimately appears in the GP record [3,4].

The lack of a significant association for breast cancer is particularly noteworthy. In Germany, mammography screening is organised nationally as a population-based programme offered to all women aged 50–75, largely independent of the referring GP, and colorectal cancer screening is likewise organised nationally. These structural features standardise the diagnostic pathway for such cancers and reduce the scope for individual GP behaviour—including any effect of physician sex—to influence what is ultimately documented, which may explain the null findings for breast and digestive cancers despite prior reports that female physicians counsel about and perform screening more frequently [9,23,24].

The magnitude of the finding should be judged in absolute terms. The difference in documented skin cancer prevalence corresponds to approximately 1.2 additional patients with a documented diagnosis per 1000 female patients and 0.8 per 1000 male patients, that is, to roughly two additional patients with a documented skin cancer diagnosis in a practice of average size. With 2.4 million patients, differences of this magnitude are estimated with high precision, and statistical significance alone is therefore not a sufficient criterion for clinical relevance. Whether a difference of this size is meaningful depends on whether it reflects earlier identification of disease or only more complete recording, which these data cannot determine.

### Strengths and Limitations

This study has several strengths. The large sample size, drawn from a representative primary care database covering a broad range of practices across Germany, provides sufficient statistical power to detect even modest associations. The use of 1:1 propensity score matching on key demographic variables reduces confounding by patient case-mix, and the analysis accounts for clustering at the practice level using both cluster-robust (GEE) and mixed-effects models, providing appropriately conservative inference. The analysis spans thirteen cancer categories, offering a comprehensive picture, and reports raw, FDR-adjusted, and Bonferroni-adjusted *p*-values to balance error control against power. The main finding was reproduced in four sensitivity analyses, including a restriction to single-physician practices in which the assignment of GP sex is unambiguous. To our knowledge, this is the first study to systematically compare documented cancer prevalence across a wide range of cancer types by GP sex in a large, real-world primary care dataset.

Several limitations must be acknowledged. First, the cross-sectional design precludes any conclusions about causality or the direction of the associations observed. Second, documented cancer prevalence reflects both the true burden of disease and the completeness and accuracy of recording, and these components cannot be disentangled in these data; the sensitivity analysis restricted to confirmed diagnoses yielded unchanged estimates, but it cannot distinguish a diagnosis established by the GP from one established elsewhere and merely recorded. Third, several potentially important patient-level confounders—including occupation, socioeconomic status, residential location, and smoking status—are not captured in the database and could not be adjusted for; healthcare utilisation was available and its inclusion did not change the results. We note that accounting for clustering at the practice level yields valid standard errors but does not remove confounding by practice-level characteristics associated with GP sex, such as location, patient structure or documentation habits, nor confounding at the patient level. Fourth, the ICD-10 categories are broad; melanoma and non-melanoma skin cancer were separated in exploratory analyses, but mesothelioma and soft-tissue sarcoma within C45–C49 were not, and heterogeneous tumour types within the remaining categories may behave differently. Fifth, specific practice-level characteristics (precise location, urban/rural status, list size) were not modelled individually, although patient volume was well-balanced (median 2564 vs. 2526 patients per practice; SMD 0.001) and practice-level heterogeneity was accounted for in the random-intercept and cluster-robust models; per-patient consultation length was not available. Sixth, the data originate from a single calendar year (2025), and temporal stability across consecutive years remains to be established. Seventh, exposure is defined by the sex of the practice owner, and individuals were assigned according to the practice they consulted, so the comparison is between patient panels rather than between randomly allocated individuals, and in group practices the physician who documents a diagnosis may be of the opposite sex; matching balanced the demographic case-mix but cannot balance the reasons why a given individual consults a given practice. Matching retained 98.8% of the smaller group but only 74.6% of the larger one, so the analysed population corresponds to the patient panels of female GPs, and unmeasured differences between practices with female and male GPs remain possible. Eighth, the mechanisms proposed here rely in part on features specific to Germany, namely direct patient access to dermatology and a dedicated billing code for statutory skin cancer screening; in systems with strict gatekeeping or without an organised skin cancer screening programme, both the magnitude and the direction of the association may differ. Finally, the IQVIA Disease Analyzer captures diagnoses as documented in practice management systems, and underreporting or inconsistent coding across practices may introduce misclassification.

## 5. Conclusions

In this large matched cross-sectional study of German primary care patients, and after accounting for clustering at the practice level, female GP sex was associated with a higher prevalence of documented skin cancer—most robustly among female patients and, in exploratory analyses, confined to non-melanoma skin cancer and to patients entitled to statutory screening—while associations for other malignancies, including mesothelial and soft tissue cancer, were not statistically significant. The higher documented skin cancer prevalence in patients of female GPs may reflect more thorough skin examination, more complete recording of externally established diagnoses, or patient-level health-seeking behaviour; these mechanisms cannot be separated with the present data, and the finding should be read as a statement about documented prevalence rather than about cancer detection. Several practical implications follow. Analysts using routine primary care or claims data to study skin cancer should consider adjusting for, or stratifying by, practice characteristics, and should account for practice-level clustering, as failure to do so produces overly narrow confidence intervals and misleading inference for rare, practice-concentrated diagnoses. Standardising skin examination and documentation practices in primary care may reduce practice-dependent variation in recorded diagnoses. The stability of results for specialist-diagnosed cancers supports the robustness of routine data for those entities. Future research should link these documentation patterns to downstream outcomes such as stage and timeliness of diagnosis to determine their clinical significance.

## Figures and Tables

**Table 1:** Baseline characteristics of the study sample after 1:1 matching.

Variable	Individuals Seen by Female GPs (n = 1,204,254)	Individuals Seen by Male GPs (n = 1,204,254)	SMD
Mean age (SD)	53.3 (19.8)	53.7 (19.8)	0.021
Age ≤ 50 years, n (%)	538,473 (44.7)	527,730 (43.8)	
Age 51–70 years, n (%)	403,360 (33.5)	406,141 (33.7)	
Age > 70 years, n (%)	262,421 (21.8)	270,383 (22.5)	
Women, n (%)	660,324 (54.8)	664,983 (55.2)	0.008
Men, n (%)	543,930 (45.2)	539,271 (44.8)	
Statutory health insurance, n (%)	1,108,274 (92.0)	1,101,487 (91.5)	0.021
Private health insurance, n (%)	95,980 (8.0)	102,767 (8.5)	
Number of individuals seen in the practice in 2025; median (IQR)	2564 (1688)	2526 (1648)	0.001
Individuals in single-physician practices, n (%)	682,378 (56.7)	1,021,240 (84.8)	0.65

Data are absolute numbers (percentages) unless otherwise specified; SMD—Standardized Mean Difference; IQR—Interquartile Range; GP—General Practitioner. The number of individuals seen refers to the practice, which is the unit of observation in the database, and not to the individual physician. Single-physician practice status was not a matching variable and is reported here to describe the practice structure of the two groups; it was addressed in a sensitivity analysis.

**Table 2:** Cluster-adjusted (GEE) association between female GP sex and the probability of documented cancer diagnoses, with raw, FDR-adjusted, and Bonferroni-adjusted *p*-values and absolute prevalence differences per 1000 patients.

Cancer Type	Patient Sex	OR (95% CI)	Raw *p*	FDR *p*	Bonferroni *p*	Absolute Difference per 1000 (95% CI)
Lip, oral cavity and pharynx	Female	1.12 (0.96–1.31)	0.136	0.625	1.000	0.07 (−0.02 to 0.16)
Lip, oral cavity and pharynx	Male	1.12 (0.98–1.28)	0.086	0.608	1.000	0.13 (−0.02 to 0.28)
Digestive organs	Female	1.00 (0.91–1.10)	0.992	0.992	1.000	0.00 (−0.45 to 0.46)
Digestive organs	Male	1.00 (0.92–1.09)	0.972	0.992	1.000	−0.01 (−0.58 to 0.55)
Respiratory and intrathoracic	Female	0.99 (0.89–1.11)	0.868	0.992	1.000	−0.02 (−0.28 to 0.23)
Respiratory and intrathoracic	Male	0.98 (0.89–1.08)	0.708	0.946	1.000	−0.06 (−0.36 to 0.24)
Bone and articular cartilage	Female	0.85 (0.63–1.16)	0.306	0.881	1.000	−0.02 (−0.06 to 0.02)
Bone and articular cartilage	Male	1.15 (0.85–1.57)	0.366	0.881	1.000	0.02 (−0.02 to 0.07)
Skin	Female	1.23 (1.11–1.35)	<0.001	0.002	0.002	1.15 (0.58 to 1.73)
Skin	Male	1.14 (1.03–1.26)	0.011	0.130	0.259	0.79 (0.18 to 1.40)
Mesothelial and soft tissue	Female	0.75 (0.43–1.31)	0.311	0.881	1.000	−0.23 (−0.72 to 0.27)
Mesothelial and soft tissue	Male	0.75 (0.45–1.25)	0.273	0.881	1.000	−0.20 (−0.59 to 0.20)
Breast	Female	1.03 (0.94–1.12)	0.538	0.881	1.000	0.30 (−0.65 to 1.24)
Female genital organs	Female	1.03 (0.93–1.14)	0.528	0.881	1.000	0.09 (−0.20 to 0.38)
Male genital organs	Male	1.00 (0.92–1.10)	0.939	0.992	1.000	0.03 (−0.85 to 0.92)
Urinary tract	Female	0.95 (0.83–1.09)	0.472	0.881	1.000	−0.09 (−0.34 to 0.16)
Urinary tract	Male	1.03 (0.93–1.13)	0.613	0.881	1.000	0.11 (−0.31 to 0.52)
Central nervous system	Female	1.06 (0.89–1.28)	0.510	0.881	1.000	0.03 (−0.06 to 0.12)
Central nervous system	Male	0.94 (0.79–1.11)	0.460	0.881	1.000	−0.04 (−0.14 to 0.06)
Thyroid and other endocrine	Female	1.13 (0.98–1.30)	0.106	0.608	1.000	0.15 (−0.03 to 0.34)
Thyroid and other endocrine	Male	0.95 (0.78–1.15)	0.613	0.881	1.000	−0.03 (−0.13 to 0.08)
Lymphoid, haematopoietic	Female	0.99 (0.87–1.12)	0.854	0.992	1.000	−0.04 (−0.43 to 0.36)
Lymphoid, haematopoietic	Male	1.02 (0.91–1.14)	0.741	0.946	1.000	0.07 (−0.35 to 0.49)

OR—Odds Ratio; CI—Confidence Interval; FDR—False Discovery Rate. Male GP sex is the reference category; an odds ratio above 1 indicates higher odds of a documented diagnosis among patients of female GPs, and a positive absolute difference indicates a higher documented prevalence among patients of female GPs. Bold values indicate the association that remained statistically significant after both FDR and Bonferroni correction. All models were estimated with generalized estimating equations accounting for clustering within practices. A mixed-model sensitivity analysis with a practice random intercept yielded consistent results.

## Data Availability

The data used in this study are subject to strict privacy and confidentiality requirements and therefore cannot be made publicly available. Legal restrictions prohibit the sharing of the raw data with third parties.

## Abbreviations

The following abbreviations are used in this manuscript:

GP	General Practitioner
OR	Odds Ratio
CI	Confidence Interval
SD	Standard Deviation
IQR	Interquartile Range
SMD	Standardized Mean Difference
ICD-10	International Classification of Diseases, 10th Revision
GEE	Generalized Estimating Equations
FDR	False Discovery Rate
ICC	Intraclass Correlation Coefficient
MOR	Median Odds Ratio
